# SIRT5 deficiency enhances the proliferative and therapeutic capacities of adipose‐derived mesenchymal stem cells via metabolic switching

**DOI:** 10.1002/ctm2.172

**Published:** 2020-09-23

**Authors:** Tiantong Ou, Wenlong Yang, Wenjia Li, Yijing Lu, Zheng Dong, Hongming Zhu, Xiaolei Sun, Zhen Dong, Xinyu Weng, Suchi Chang, Hua Li, Yufan Li, Zhiwei Qiu, Kai Hu, Aijun Sun, Junbo Ge

**Affiliations:** ^1^ Department of Cardiology, Zhongshan Hospital Fudan University, Shanghai Institute of Cardiovascular Diseases Shanghai China; ^2^ Institute of Biomedical Sciences Fudan University Shanghai China; ^3^ Translational Medical Center for Stem Cell Therapy & Institute for Regenerative Medicine, Shanghai East Hospital Tongji University School of Medicine Shanghai China

**Keywords:** adipose‐derived mesenchymal stem cells, cell proliferation, hind limb ischemia, metabolic switching, SIRT5

## Abstract

**Background:**

Mesenchymal stem cells (MSCs) have therapeutic potential for multiple ischemic diseases. However, in vitro expansion of MSCs before clinical application leads to metabolic reprogramming from glycolysis to oxidative phosphorylation, drastically impairing their proliferative and therapeutic capacities. This study aimed to define the regulatory effects of Sirtuin 5 (SIRT5) on the proliferative and therapeutic functions of adipose‐derived MSCs (ADMSCs) during in vitro expansion.

**Methods:**

ADMSCs were isolated from wild‐type (WT) and Sirt5‐knockout (Sirt5^−/−^) mice. Cell counting assay was used to investigate the proliferative capacities of the ADMSCs. Dihydroethidium and senescence‐associated β‐galactosidase stainings were used to measure intracellular ROS and senescence levels. Mass spectrometry was used to analyze protein succinylation. Oxygen consumption rates and extra cellular acidification rates were measured as indicators of mitochondrial respiration and glycolysis. Metabolic‐related genes expression were verified by quantitative PCR and western blot. Hind limb ischemia mouse model was used to evaluate the therapeutic potentials of WT and Sirt5^−/−^ ADSMCs.

**Results:**

SIRT5 protein levels were upregulated in ADMCs during in vitro expansion. Sirt5^−/−^ ADMSCs exhibited a higher proliferation rate, delayed senescence, and reduced ROS accumulation. Furthermore, elevated protein succinylation levels were observed in Sirt5^−/−^ ADMSCs, leading to the reduced activity of tricarboxylic acid cycle‐related enzymes and attenuated mitochondrial respiration. Glucose uptake, glycolysis, and pentose phosphate pathway were elevated in Sirt5^−/−^ ADMSCs. Inhibition of succinylation by glycine or re‐expression of Sirt5 reversed the metabolic alterations in Sirt5^−/‐^ ADMSCs, thus abolishing their enhanced proliferative capacities. In the hind limb ischemia mouse model, SIRT5^−/−^ ADMSCs transplantation enhanced blood flow recovery and angiogenesis compared with WT ADMSCs.

**Conclusions:**

Our results indicate that SIRT5 deficiency during ADMSC culture expansion leads to reversed metabolic pattern, enhanced proliferative capacities, and improved therapeutic outcomes. These data suggest SIRT5 as a potential target to enhance the functional properties of MSCs for clinical application.

## Graphical abstract

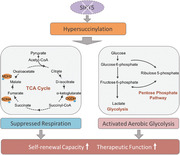

## 1 BACKGROUND

Mesenchymal stem cells (MSCs) are typical adult stem cells that possess the ability to promote tissue repair through tissue‐specific differentiation, paracrine effects, and immunomodulation.^1^ MSCs have been extensively studied in both basic research studies as well as clinical trials for ischemic diseases because these cells are easily isolated, non‐immunogenic, and exhibit great therapeutic potential.^2^, ^3^ Primary MSCs are scarce and culture expansion is essential before clinical application. However, the in vitro artificial microenvironment may reprogram the energy metabolism of MSCs, which may lead to the disruption of homeostasis, exhaustion of their self‐renewal capacities, and eventual impairment of their therapeutic efficacy.^4^, ^5^ Therefore, providing MSCs with sufficient therapeutic potential and maintaining their proliferative capacity during in vitro expansion are crucial for clinical outcomes.^6^

Under in vivo conditions, the low oxygen tension in the stem cell niche stabilizes hypoxia‐inducible factor‐1α (HIF‐1α) and maintains high glycolysis levels with low mitochondrial activity in MSCs^7^, ^8^ This phenomenon of modified cellular metabolism in certain cell types is known as the Warburg effect. Although glycolysis seems “less efficient” compared to oxidative phosphorylation (OXPHOS), this particular metabolic process produces necessary intermediates for proliferation and minimizes the production of harmful reactive oxygen species (ROS) from mitochondria.^9^ However, after isolation, MSCs are exposed to standard culture conditions, which have a higher oxygen tension (21%). An altered oxygen concentration may lead to proteasome degradation of HIF‐1α and a metabolic shift from glycolysis to OXPHOS during in vitro expansion. ROS accumulation may also occur due to mitochondrial activation. Culture‐induced metabolic reprogramming may eventually lead to impaired proliferation and accelerated senescence.^5^, ^10^ Therefore, enhancing in vitro aerobic glycolysis, either by manipulating the culture microenvironment or by targeting metabolic‐related genes, promotes the self‐renewal capacities and preserves the physiological functions of stem cells.^2^, ^10^, ^11^

Sirtuin (SIRT) family members are classified as class III histone deacetylases and are master regulators of posttranslational modifications^12^, ^13^, ^14^, ^15^ that target diverse protein substrates involved in multiple metabolic pathways.^14^, ^16^, ^17^, ^18^ SIRT5 is among these SIRT family members and possesses various enzymatic activities, including deacetylation, desuccinylation, demalonylation, and deglutarylation. Through these protein modifications, SIRT5 regulates diverse metabolic processes that involve glycolysis, OXPHOS, fatty acid oxidation, and the urea cycle.^19^ Although the regulatory effects of SIRT5 are highly context specific in different cell types, changes in SIRT5 levels generally cause metabolic shifts and cell function alterations.^20^, ^21^, ^22^ However, its metabolic control and functional effects in MSCs during in vitro expansion remain unclear. In this study, we found that SIRT5 levels changed significantly in adipose‐derived mesenchymal stem/stromal cells (ADMSCs) under in vitro culture conditions. SIRT5 deficiency resulted in altered succinylation levels of tricarboxylic acid (TCA) cycle‐related enzymes, which substantially altered the metabolic pattern and proliferative capacity of ADMSCs and led to enhanced therapeutic efficacy in a hind limb ischemia model.

## 2 METHODS

### 2.1 Animals

Sirt5‐knockout mice were kindly provided by Professor Hongxiu Yu (Institutes of Biomedical Sciences, Fudan University, Shanghai, China). The mice were housed in a pathogen‐free facility and were given a standard mouse chow diet and tap water. All experimental animal protocols were approved by the University Committee on the Care and Use of Animal.

### 2.2 Cell isolation and culture

ADMSCs were isolated from subcutaneous fat deposits of 4‐5‐week‐old male Sirt5‐knockout mice or littermate control C57BL/6 mice, as previously described.^23^ Inguinal subcutaneous adipose tissues were cut and finely minced in phosphate‐buffered saline (PBS). The tissue samples were then transferred into a solution of 0.1% type I collagenase in PBS and incubated at 37°C for 1 h with agitation. The digested tissues were then centrifuged. After aspiration of the floating mature adipocytes, the pellets were re‐suspended and filtered through a 70‐μm cell strainer (BD Falcon, San Jose, CA, USA). Cells were plated into 10‐cm dishes in C57BL/6 mouse adipose‐derived mesenchymal stem cell basal medium (Cyagen, Suzhou, China).

### 2.3 Western blot analysis

ADMSCs were lysed in radioimmunoprecipitation assay (RIPA) buffer with Halt Protease Inhibitor Cocktail (WeiAo, Shanghai, China). Protein concentrations were measured using a BCA Protein Assay kit (WeiAo). Protein samples were separated by 10% or 12% gel electrophoresis, transferred to a poly‐vinylidene fluoride membrane, and blocked with 5% bovine serum albumin (BSA) for 2 h. The membrane was incubated at 4°C overnight with primary antibodies and then incubated at room temperature for 2 h with secondary antibodies. The following antibodies were used: anti‐SIRT5 (Cell Signaling Technology, Danvers, MA, USA; 8782S; 1:1000), anti‐acetylated lysine (Cell Signaling Technology; 9441S; 1:1000), anti‐succinylated lysine (PTM Biolab, Hangzhou, China; PTM419 1:1000; PTM401 1:1000;), anti‐malonylated lysine (PTM Biolab; PTM901 1:1000), anti‐glutarylated lysine (PTM Biolab; PTM1152 1:1000), anti‐SDHA (Santa Cruz Biotechnology, Dallas, Texas, USA; 166909 1:1000 for WB, 1:50 for IP), anti‐OGDH (Cell Signaling Technology; 26865S 1:1000 for WB, 1:50 for IP), anti‐MDH2 (Abcam, Cambridge, UK; 181857 1:10000 for WB; 110317 1:50 for IP;), anti‐GLUT1 (Abcam; 115730 1:1000), anti‐HIF‐1α (Abcam; 16066 1:1000), anti‐PKM2 (Cell Signaling Technology; 4053S 1:1000), anti‐G6PD (Proteintech Rosemont, IL, USA; 66373 1:1000), anti‐PGD (Proteintech; 14718 1:1000), and anti‐TKT (Cell Signaling Technology; 8616S 1:1000).

### 2.4 Quantitative PCR

RNA was extracted from cells using RNAiso Plus (Takara, Beijing, China). Complementary DNA (cDNA) was synthesized from 1000 ng total RNA using PrimeScript RT Master Mix (Takara, RR036A) and a Bio‐Rad T100 Thermal Cycler. Quantitative PCR (qPCR) was performed in triplicate as 10 μL reactions for each sample using Hieff qPCR SYBR Green Master Mix (Yeasen, Shanghai, China). Primers were purchased from TsingKe Biological Technology (Beijing, China). β‐Actin was used as an internal control.

### 2.5 Cell counting proliferation assay

Cells were detached with 0.25% trypsin, suspended in stem cell basal medium, and counted using a hemocytometer. An equal number of cells (1 × 10^4^) was seeded into the individual wells of 12‐well plates. The total number of cells per well was determined at days 2, 4, and 6 post‐seeding.

### 2.6 Senescence‐associated β‐galactosidase staining

Senescence‐associated β‐galactosidase (SA‐β‐gal) staining was performed using the Senescence β‐Galactosidase Staining Kit (Cell Signaling Technology). Briefly, cells were cultured in a 35‐mm plate until 90% confluence, rinsed with PBS, and fixed with a fixative solution. Cells were then incubated overnight at 37°C in β‐galactosidase‐staining solution. Cell were then observed under a microscope for blue coloring while the β‐galactosidase solution was still in the plate.

### 2.7 Oxygen consumption rate and extracellular acidification rate measurements

MSCs were seeded into XF 96‐well microplates (40,000 cells/well) and incubated overnight in a 37°C incubator with 5% CO_2_. The oxygen consumption rates (OCRs) and extracellular acidification rates (ECARs) were measured by an XF96 Extracellular Flux Analyzer (Seahorse Biosciences, Agilent, CA, USA) according to the manufacturer's protocol.

### 2.8 Enzymatic activity assay

Enzyme activities of MDH, SDH, and OGDH were determined using Enzymatic activity assay kits (Solarbio, Beijing, China. SOL‐BC1040, SOL‐BC0710, SOL‐BC0950) according to the manufacturer's protocol.

### 2.9 Glucose uptake assay

WT and Sirt5^−/−^ ADMSCs were seeded into a 96‐well plate (40 000 cells/well). Cells were incubated in glucose‐free medium for 30 min, followed by incubation in culture medium containing 600 mM fluorescent tracer 2‐NBDG (Sigma, Saint Louis, MO, USA) and 3.3 mM glucose for another 30 min at 37°C. The cellular uptake of 2‐NBDG was measured using a florescent microplate reader (Biotek, Winooski, VT, USA).

### 2.10 2,7‐Dichlorodi‐hydrofluorescein diacetate (DCFH‐DA) staining

ADMSCs were seeded into a six‐well plate and cultured until 60‐70% confluence. Before staining, ADMSCs in H_2_O_2_ groups were treated with 200 μM H_2_O_2_ for 12 h. Cells were then detached with 0.25% trypsin and stained with DCFH‐DA (Yeasen, Shanghai, China, 50101ES01) according to the manufacturer's protocol. ROS accumulation was measured by flow cytometry using BD Arial III flow cytometer.

### 2.11 Sirt5 re‐expression in Sirt5^−/−^ ADMSCs

Sirt5^−/−^ was transfected by Sirt5 overexpression lentivirus (Hanyin Biotechnology Limited Company, shanghai, China, HY‐KL‐00752) or control lentivirus. After 12 h transfection, cells were then screened by puromycin for another 3 days before further uses.

### 2.12 Hind limb ischemia model and ADMSC transplantation

The hind limb ischemia model was established as previously reported.^24^ Briefly, 8‐week‐old male C57BL/6 mice (n = 5‐6/group) were anesthetized by intraperitoneal ketamine injection (80 μg/g body weight). A skin incision was made to access the femoral artery, which was then isolated from the femoral vein and nerve. Next, the artery was ligated at the proximal to superficial epigastric artery and distal to the bifurcation of the saphenous and popliteal arteries. After surgery, WT or Sirt5^−/−^ ADMSCs (10^6^ cells in 150 μL normal saline [NS]), or an equal volume of NS for controls, were intramuscularly injected into the thigh muscles of the mice around the ligation sites.

### 2.13 Laser Doppler perfusion imaging

Hind limb perfusion was evaluated by Laser Doppler perfusion imaging (PeriScan PIM3 system, Perimed, Sweden). Mice were anesthetized and placed on a heating pad (37°C) for 5 min before imaging analysis. Blood flow levels were quantified by average relative units of flux from the knee to toe using PIMsoft software (Perimed med, Sweden). Perfusion ratios were calculated as ischemic limbs versus non‐ischemic limbs.

### 2.14 In vivo cell tracking

Before transplanted to ischemic limb, WT and Sirt5^−/−^ ADMSCs were harvested and labeled with DiR Iodide (Yeasen, 40757ES25). Three and 7 days after cell delivery, mice were anesthetized and fluorescence imaging was obtained using IVIS SpectrumCT In Vivo Imaging System (PerkinElmer, Waltham, MA, USA).

### 2.15 Statistical analyses

Statistical analyses were performed using GraphPad Prism 7 (San Diego, CA, USA). Data are expressed as the mean values ± SD. Two‐tailed Student's *t*‐tests were performed to analyze differences between two groups. *F*‐tests were used to compare variances. A *P*‐value < .05 was considered to indicate statistical significance.

## 3 RESULTS

### 3.1 Elevated SIRT5 levels were detected in ADMSCs during in vitro expansion

**FIGURE 1:**
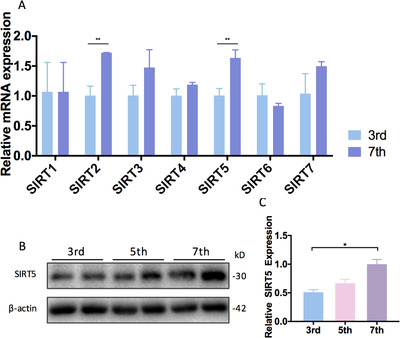
Elevated SIRT5 levels were detected in ADMSCs during in vitro expansion. A, Relative mRNA expression levels of Sirt1–7 in passages 3 and 7 of wild‐type ADMSCs (n = 3). B,C, Western blot analysis and expression level quantification of SIRT5 in passages 3, 5, and 7 of wild‐type ADMSCs (n = 3). Data are expressed as the mean ± SD. ^*^ *P* < .05; ^**^ *P* < .01

ADMSCs were isolated from the subcutaneous fat of C57BL/6 mice and were sorted and evaluated for differentiation potential by expression of MSC markers, including CD29, CD105, and SCA‐1, as well as the absence of hematopoietic lineage marker CD45 (Figure S1A,B). During continuous *in vitro* passaging, late‐passage ADMSCs (passage 7‐8) showed a flattened cell morphology, which is a sign of senescence, compared with early‐passage ADMSCs (passage 3‐4). Therefore, the *Sirt 1–7* mRNA expression levels were tested in both early‐ and late‐passage ADMSCs (Figure 1A). Interesting, *Sirt5* mRNA levels were significantly altered during late‐stage culture expansion. Western blot analysis further confirmed upregulated SIRT5 protein levels in late‐passage ADMSCs (Figure 1B,C), strongly suggested a regulatory effect of SIRT5 on in vitro cultured ADMSCs.

### 3.2 Sirt5‐knockout ADMSCs exhibited sustained proliferation with reduced ROS accumulation and delayed senescence

**FIGURE 2:**
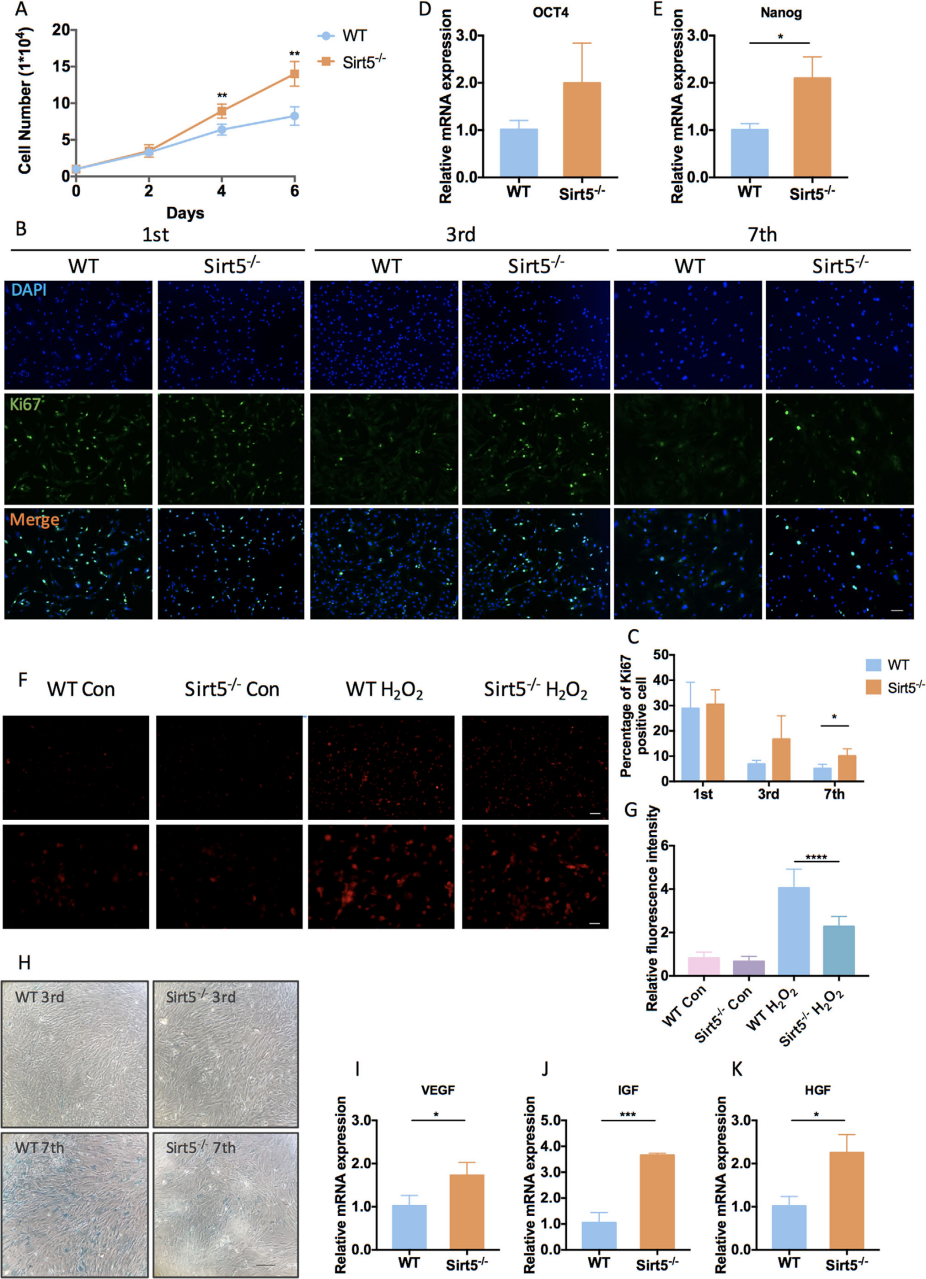
SIRT5‐knockout ADMSCs exhibited sustained self‐renewal capacities with reduced ROS accumulation and delayed senescence. A, Cell counting assay of wild‐type (WT) and Sirt5‐knockout (Sirt5^−/−^) ADMSCs at days 2, 4, and 6 after seeding (n = 4). B, Ki67 staining of WT and Sirt5^−/−^ ADMSCs at passages 1, 3, and 7. Scale bar: 100 μm. C, Percentages of WT and Sirt5^−/−^ Ki67^+^ ADMSCs at passages 1, 3, and 7 (n = 4, different fields). D,E, Relative mRNA expression levels of multipotent stem cell markers (n = 3). F, Dihydroethidium (DHE) staining (red) of WT and Sirt5^−/‐^ ADMSCs under basal conditions (control) and after 12 h of H_2_O_2_ (200 μM) treatment. Scale bars: upper panel, 100 μm; lower panel, 50 μm. G, Relative fluorescence intensities of DHE staining (n = 5–8, different fields). H, Senescence‐associated β‐galactosidase (SA‐β‐gal) staining (blue) of WT and Sirt5^−/−^ ADMSCs at passages 3 and 7 (upper panel: passage 3; lower panel: passage 7). Scale bar: 200 μm. I‐K, Relative mRNA expression levels of multi‐growth factors (n = 3). Data are expressed as the mean ± SD. ^*^ *P* < .05; ^**^ *P* < .01; ^***^ *P* < .001; ^****^ *P* < .0001. Abbreviations: OCT4, octamer‐binding protein 4; VEGF, vascular endothelial growth factor; IGF, insulin‐like growth factor; HGF, hepatocyte growth factor

To explore the effects of SIRT5 in ADMSCs during in vitro expansion, Sirt5^−/−^ and WT control ADMSCs were isolated from Sirt5‐knockout and WT mice, respectively. Downregulated SIRT5 protein levels were confirmed by western blot (Figure S1C). SIRT5 deficiency did not affect cell marker expression or impair the multi‐lineage differentiation of the ADMSCs (Figure S1A,B). A cell counting assay showed that Sirt5^−/−^ ADMSCs had a significantly higher proliferation rate than WT ADMSCs (Figure 2A). Population doubling time also confirmed an enhanced proliferation phenotype of Sirt5^−/−^ ADMSCs (Figure S2A). Immunofluorescence analysis of cellular proliferative marker Ki67 revealed that the ratio of Ki67^+^ cells was similar between Sirt5^−/‐^ and WT ADMSCs at passage 1. However, a higher ratio of Ki67^+^ Sirt5^−/‐^ ADMSCs was detected at passages 3 and 7 compared with WT ADMSCs (Figure 2B,C). These results suggest that SIRT deficiency results in remarkable retention of self‐renewal capacities in ADMSCs. Furthermore, SIRT5 deficiency led to a twofold increase in Nanog and Oct4 expression, indicating better preservation of stemness (Figure 2D,E). Because cellular senescence and high oxygen tension‐induced oxidative stress also compromise self‐renewal capacities,^25^ cell senescence and ROS levels were examined in ADMSCs. Under basal conditions, cellular ROS levels were relatively low in both Sirt5^−/−^ and WT ADMSCs. After 12 h of H_2_O_2_ treatment, the ROS levels increased in both cell types; however, Sirt5^−/−^ ADMSCs exhibited less ROS accumulation than WT ADMSCs (Figure 2F,G). Additionally, a reduction in SA‐β‐gal accumulation was observed at passage 7 in Sirt5^−/−^ ADMSCs compared with WT ADMSCs (Figure 2H), indicating delayed senescence in Sirt5^−/−^ ADMSCs during in vitro expansion. The mRNA expressions of classical senescence associated secretory phenotypes (SASP) and aging‐related markers were also attenuated by SIRT5 deficiency (Figure S2B). Moreover, SIRT5 deficiency led to increased mRNA expression of vascular endothelial growth factor (Vegf), insulin‐like growth factor (Igf), and hepatocyte growth factor (Hgf) in ADMSCs, further supporting the enhanced proliferative capacities of Sirt5^−/−^ ADMSCs (Figure 2I–K). Altogether, these data demonstrate the maintenance of stemness and self‐renewal capacities in Sirt5^−/−^ ADMSCs during in vitro expansion.

### 3.3 Metabolic‐related proteins were hypersuccinylated in SIRT5‐knockout ADMSCs

**FIGURE 3:**
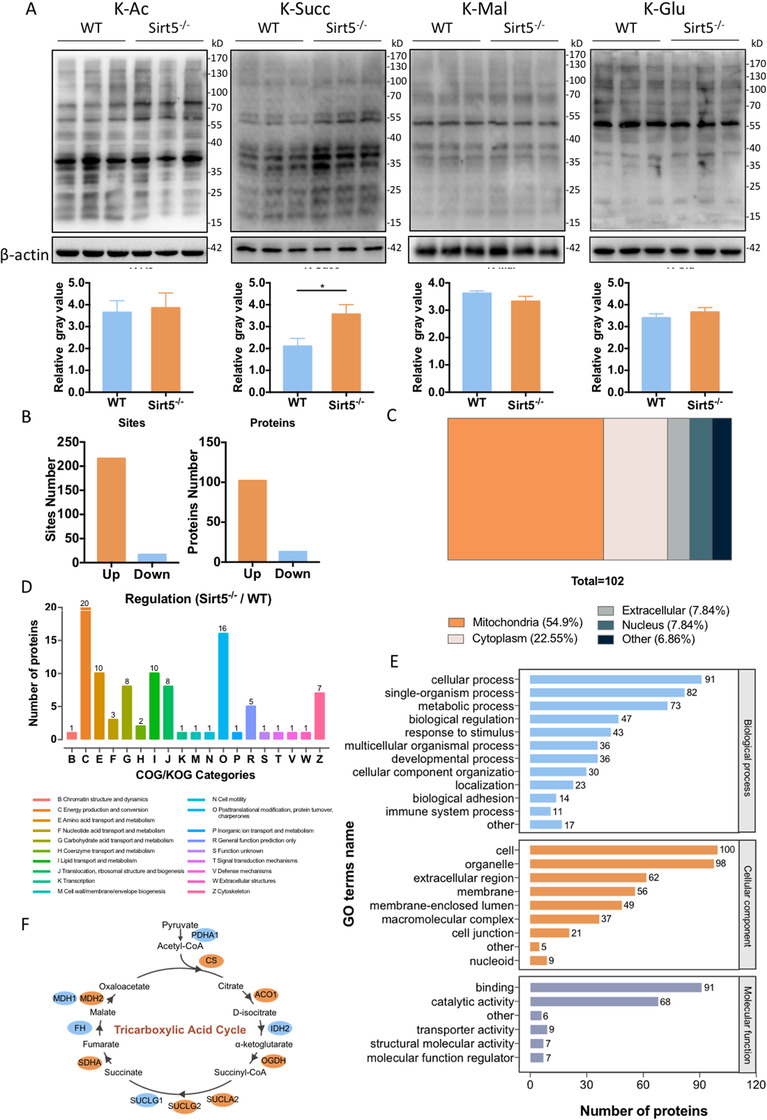
Metabolic‐related proteins were hypersuccinylated in SIRT5‐knockout ADMSCs. A, Lysine acetylation (Kac), succinylation (Ksucc), malonylation (Kmal), and glutarylation (Kglu) levels of wild‐type (WT) and Sirt5‐knockout (Sirt5^−/−^) ADMSCs. B, Differentially succinylated proteins and sites in WT and Sirt5^−/−^ ADMSCs. C, Localization of hypersuccinylated proteins in Sirt5^−/−^ versus WT ADMSCs. D, COG/KOG categories of hypersuccinylated proteins in Sirt5^−/−^ versus WT ADMSCs. E, GO terms of hypersuccinylated proteins in Sirt5^−/−^ versus WT ADMSCs. F, KEGG pathway enrichment analysis of succinylated enzymes that are involved in the TCA cycle. Blue oval: unchanged proteins. Orange oval: succinylation level increased more than twofold in Sirt5^−/−^ versus WT ADMSCs

Next, we aimed to explore the underlying mechanism of the enhanced proliferation in Sirt5^−/−^ ADMSCs. Because SIRT5 possesses lysine deacetylation, desuccinylation, demalonylation, and deglutarylation functions, the levels of these posttranslational modifications were evaluated in WT and Sirt5^−/−^ ADMSCs (Figure 3A; Figure S3A). Compared to WT ADMSCS, acetylation, malonylation, and glutarylation levels were not markedly altered by SIRT5 deficiency; however, succinylation was increased in Sirt5^−/−^ ADMSCs. To identify lysine‐succinylated (Ksucc) sites and proteins regulated by SIRT5, the Ksucc proteome between WT and Sirt5^−/−^ ADMSCs was quantified using HPLC‐MS/MS. Among 115 proteins, 233 succinylation sites were identified from these cells, and most of these proteins (216 sites of 102 proteins) were hypersuccinylated in Sirt5^−/−^ ADMSCs (Figure 3B). Over 50% of these hypersuccinylated proteins were localized to mitochondria, which is consistent with the cellular distribution of SIRT5^16^ (Figure 3C). To further evaluate the biological functions of these hypersuccinylated proteins, enrichment analysis was performed with Gene Ontology annotation database (GO) and Clusters of Orthologous Groups of proteins (COGs/KOG category description) (Figure 3D,E). Consistent with previous reports, SIRT5 deficiency led to the enrichment of hypersuccinylated proteins involved in cellular metabolic processes as well as energy production and conversion. Furthermore, KEGG pathway enrichment analysis showed that over half of the TCA cycle enzymes were hypersuccinylated in Sirt5^−/−^ ADMSCs (Figure 3F). These proteomics data collectively indicate that protein hypersuccinylation in response to SIRT5 deficiency elicits a metabolic regulatory effect in ADMSCs.

### 3.4 SIRT5 deficiency reduced the catalytic activities of hypersuccinylated TCA cycle‐related enzymes and suppressed oxidative phosphorylation

**FIGURE 4:**
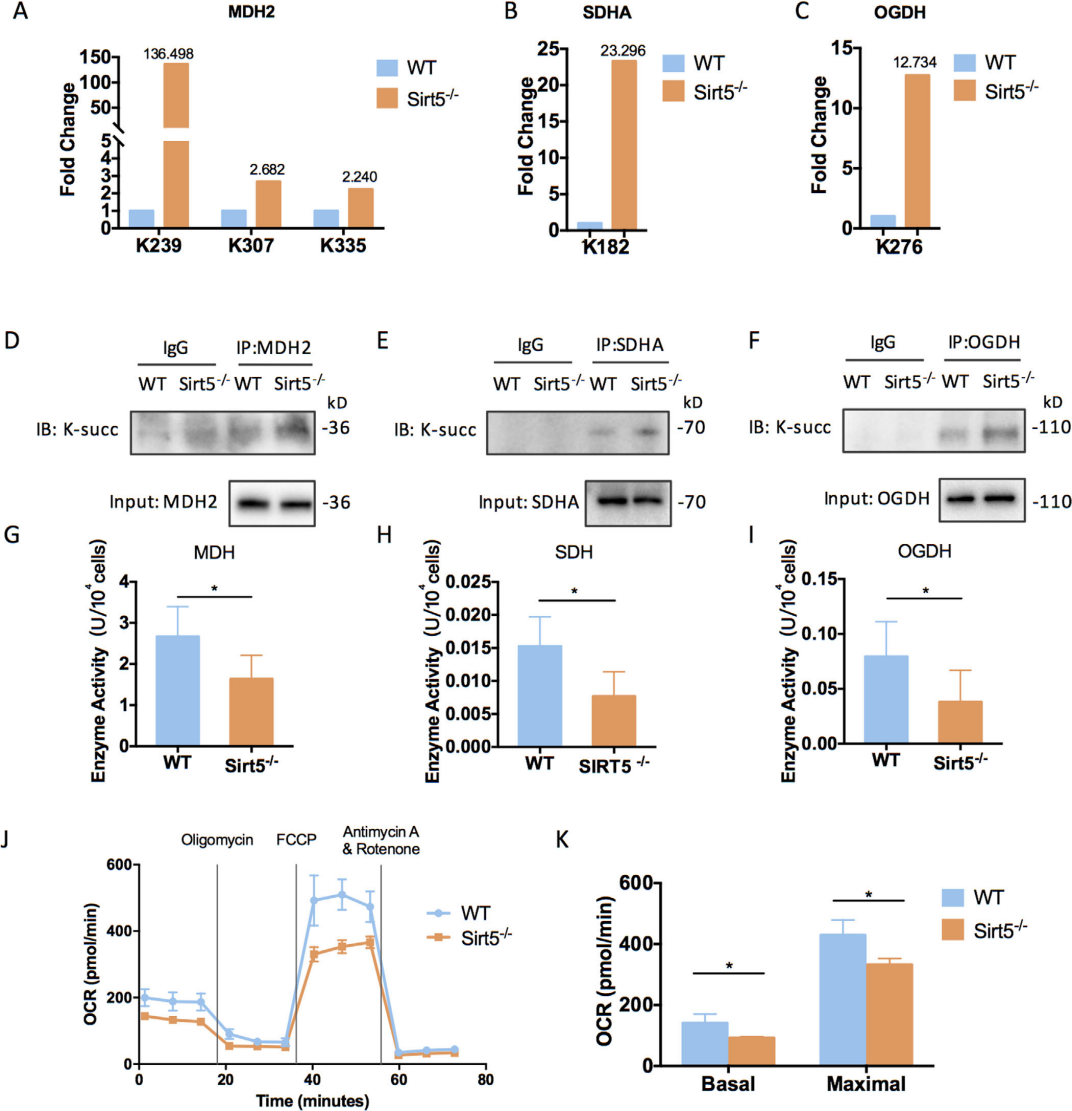
SIRT5 deficiency reduced the catalytic activities of hypersuccinylated TCA cycle‐related enzymes and suppressed oxidative phosphorylation. A–C, Fold changes of lysine succinylation sites of MDH2 (A), SDHA (B), and OGDH (C) in wild‐type (WT) and Sirt5‐knockout (Sirt5^−/−^) ADMSCs. D–F, Succinylation levels of TCA cycle‐related enzymes in WT and Sirt5^−/−^ ADMSCs: MDH2 (D), SDHA (E), and OGDH (F). G–I, Catalytic activities of TCA cycle‐related enzymes in WT and Sirt5^−/−^ ADMSCs: MDH2 (G), SDHA (H), and OGDH (I) (n = 5). J, Oxygen consumption rates (OCRs) of WT and Sirt5^−/−^ ADMSCs. (K) Basal and maximal respiration rates of WT and Sirt5^−/‐^ ADMSCs (n = 4). Data are expressed as the mean ± SD. ^*^ *P* < .05. Abbreviations: MDH2, malate dehydrogenase 2; OGDH, 2‐oxoglutarate dehydrogenase; SDHA, succinate dehydrogenase subunit A

The metabolic balance between glycolysis and OXPHOS is of great importance to the self‐renewal capacities and therapeutic functions of ADMSCs during in vitro expansion.^5^ Therefore, the metabolic patterns were evaluated in WT and Sirt5^−/−^ ADMSCs. According to our proteomics data, the most highly succinylated TCA cycle proteins were succinate dehydrogenase subunit A (SDHA), malate dehydrogenase 2 (MDH2), and 2‐oxoglutarate dehydrogenase (OGDH) (Figure 4A–C). To examine the effect of SIRT5 deficiency on these metabolic proteins, immunoprecipitation assay was used to verify the hypersuccinylation status of the TCA cycle‐related enzymes. In line with our proteomics results, elevated succinylation levels of SDHA, MDH2, and OGDH were detected in Sirt5^−/−^ ADMSCs (Figure 4D–F). Modification of these proteins by succinylation led to their significantly attenuated enzymatic activities, which indicated suppressed aerobic respiration (Figure 4G–I). Oxygen consumption rates (OCRs) were measured as an indicator of mitochondrial function, and a reduced OCR was confirmed in the Sirt5^−/−^ ADMSCs (Figure 4J). Furthermore, Sirt5^−/−^ ADMSCs exhibited a 28% reduction in their basal respiration rate and a 22% reduction in their maximal respiration (Figure 4K). In conclusion, hypersuccinylation of TCA cycle‐related enzymes significantly attenuated OXPHOS in Sirt5^−/−^ ADMSCs compared with WT ADMSCs.

### 3.5 Aerobic glycolysis and metabolism via the pentose phosphate pathway were enhanced in Sirt5‐knockout ADMSCs

**FIGURE 5:**
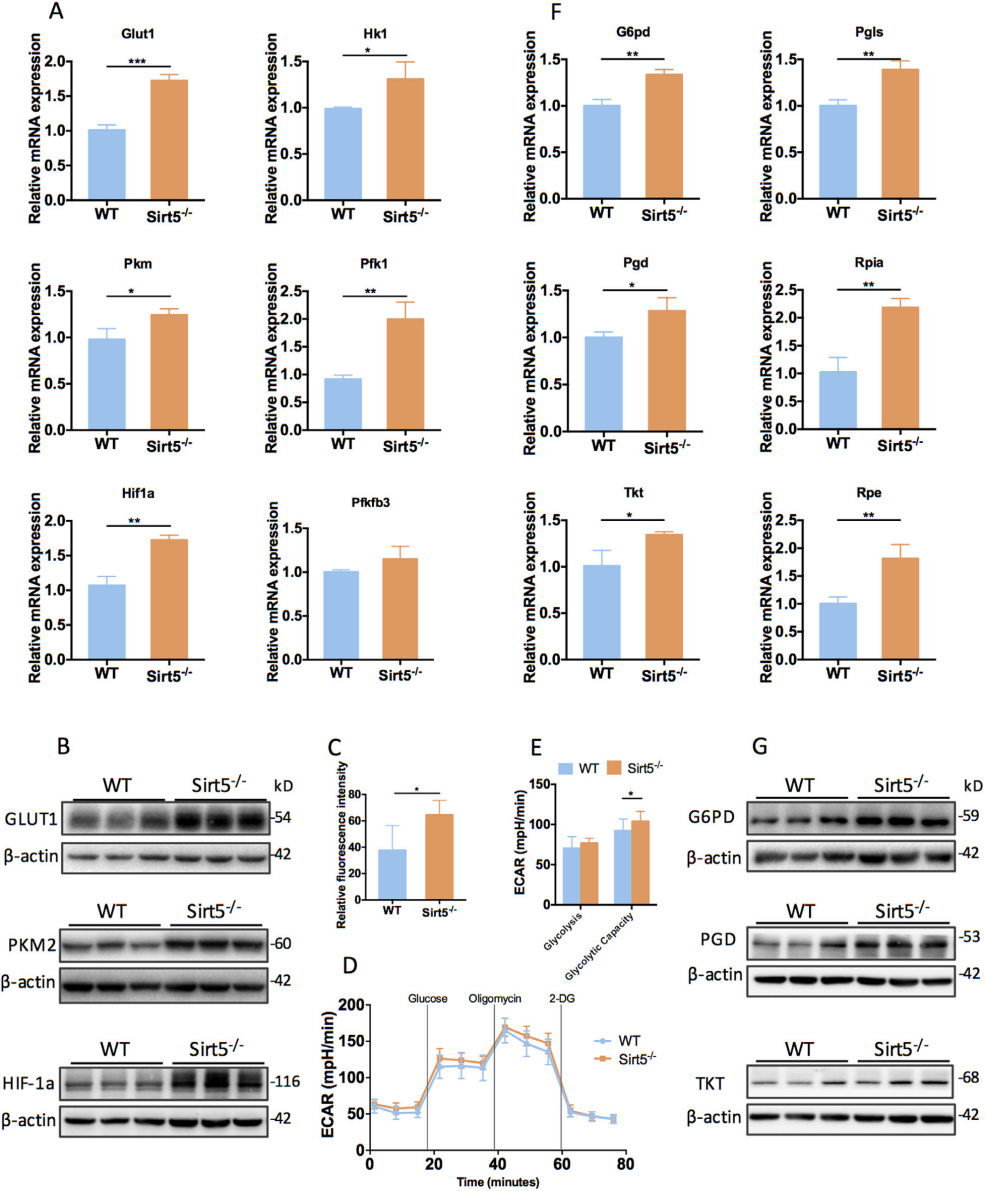
Aerobic glycolysis and metabolism via the pentose phosphate pathway were enhanced in Sirt5‐knockout ADMSCs. A, Relative mRNA expression levels of glycolysis‐related genes (n = 3). B, Protein expression levels of glycolytic enzymes. C, Quantification of glucose intake (n = 8). D, Extracellular acidification rates (ECARs) of wild‐type (WT) and Sirt5‐knockout (Sirt5^−/−^) ADMSCs. E, Basal glycolysis and glycolytic capacities of WT and Sirt5^−/‐^ ADMSCs (n = 12). F, Relative mRNA expression levels of oxidative (*G6pd, Pgls, Pgd*) and nonoxidative (*Rpia, Tkt, Rpe*) pentose phosphate pathway‐related genes (n = 3). G, Protein expression levels of pentose phosphate pathway‐related enzymes. Data are expressed as the mean ± SD. ^*^ *P* < .05; ^**^ *P* < .01; ^***^ *P* < .001; ^****^ *P* < .0001. Abbreviations: G6PD, glucose‐6‐phosphate dehydrogenase; GLUT1, glucose transporter 1; HIF‐1α, hypoxia‐inducible factor‐1‐alpha; HK1, hexokinase 1; PFK1, 6‐phosphofructokinase 1; PFKfb3, 6‐phosphofructo‐2‐kinase/fructose‐2,6‐biphosphatase 3; PGD, 6‐phosphogluconate dehydrogenase; PGLS, 6‐phosphogluconolactonase; PKM2, pyruvate kinase M2; RPE, ribulose‐5‐phosphate 3‐epimerase; RPIA, ribose‐5‐phosphate isomerase; TKT, transketolase

Because Sirt5^−/−^ ADMSCs exhibited decreased mitochondrial respiration, we evaluated other possibly altered glucose metabolic pathways in these cells. We examined the expression levels of glycolysis‐related factors in WT and Sirt5^−/−^ ADMSCs and found that the mRNA levels of glycolysis‐related genes were significantly elevated in Sirt5^−/−^ ADMSCs (Figure 5A). Among these genes, we identified elevated expression levels of glucose transporter 1 (*Glut1*), pyruvate kinase isozyme M2 (*Pkm2*), and *Hif‐1α*, which all have important roles in aerobic glycolysis in highly proliferative cells. Western blot analysis further confirmed the upregulated protein expression levels of these factors in Sirt5^−/−^ ADMSCs (Figures 5B; Figure S4A). As expected, elevated GLUT1 expression also led to increased glucose uptake in Sirt5^−/−^ ADMSCs (Figure. 5C). However, Seahorse metabolic analysis revealed similar ECARs under basal glycolysis conditions in both cell types in addition to a slight increase (13%) in the maximal glycolysis capacity of Sirt5^−/−^ ADMSCs (Figure. 5D,E), indicating that glucose was not being completely metabolized into lactic acid via glycolysis, despite increased glucose uptake in Sirt5^−/−^ ADMSCs. The pentose phosphate pathway (PPP) is connected to glycolysis and has been reported to enhance tumor cell proliferation.^26^ Therefore, we evaluated the expression levels of PPP‐related enzymes and found that key enzymes of the oxidative metabolic branch (glucose‐6‐phosphate dehydrogenase [G6PD], 6‐phosphgluconolactonase [PGLS], and 6‐phosphogluconate dehydrogenase [PGD] as well as the nonoxidative metabolic branch (ribose‐5‐phosphate isomerase [RPIA], ribulose‐5‐phosphate 3‐epimerase [RPE], and transketolase [TKT]) were both upregulated in Sirt5^−/−^ ADMSCs (Figure 5F,G; Figure S4B). Altogether, these results indicate elevated aerobic glycolysis and metabolism via the PPP in Sirt5^−/−^ ADMSCs.

### 3.6 Inhibition of succinylation by glycine reversed metabolic alterations and reduced proliferation in Sirt5‐knockout ADMSCs

**FIGURE 6:**
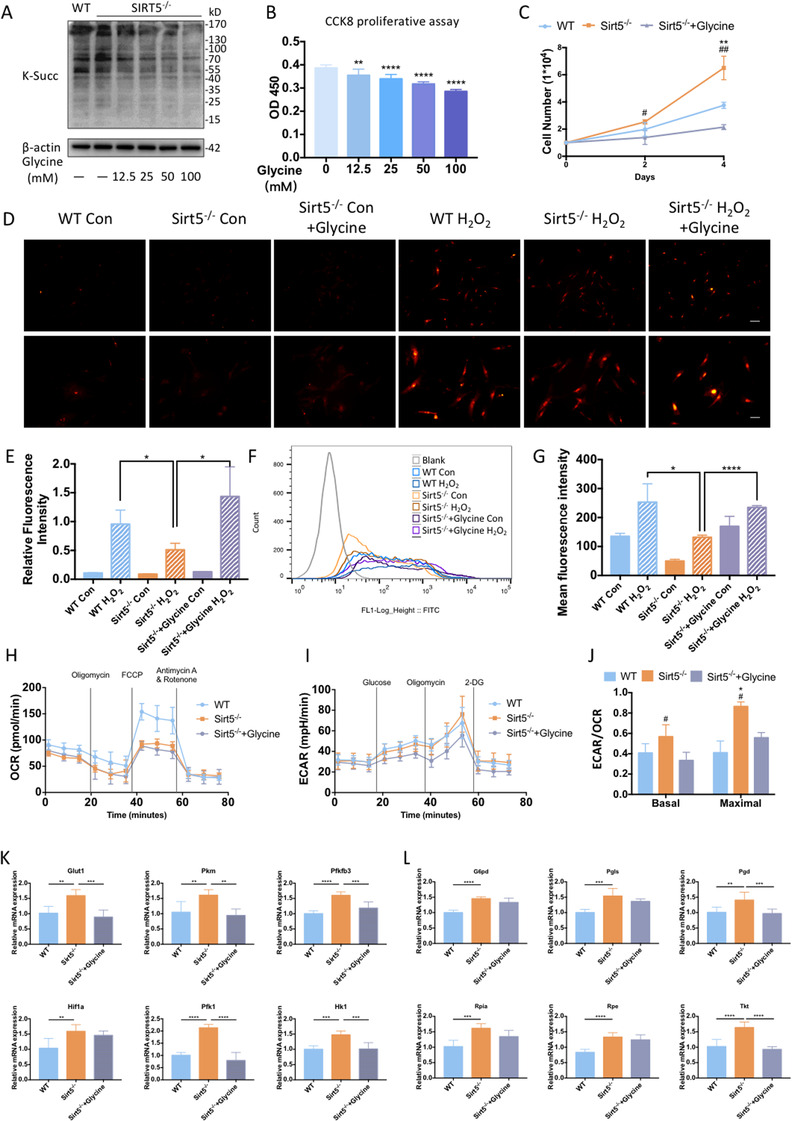
Inhibition of succinylation by glycine reversed metabolic alterations and reduced proliferation in Sirt5‐knockout ADMSCs. A, Lysine succinylation levels in wild‐type (WT) ADMSCs, Sirt5‐knockout (Sirt5^−/−^) ADMSCs, and Sirt5^−/−^ ADMSCs treated with different concentrations of glycine. B, CCK8 cell viability assay in ADMSCs 24 h after seeding (n = 8). C, Cell counting assay of WT ADMSCs, Sirt5^−/−^ ADMSCs, and glycine‐treated (25 mM) Sirt5^−/−^ ADMSCs at days 2 and 4 after seeding (n = 3). D, Dihydroethidium (DHE) staining (red) of WT ADMSCs, Sirt5^−/−^ ADMSCs, and glycine‐treated (25 mM) Sirt5^−/−^ ADMSCs under basal conditions and after 12 h of H_2_O_2_ (200 μM) treatment. Scale bars: upper panel, 100 μm; lower panel, 50 μm. E, Relative fluorescence intensities of DHE staining (n = 3, different fields). F, Representative flow cytometry assay image (DCFH‐DA staining) of WT ADMSCs, Sirt5^−/−^ ADMSCs, and glycine‐treated (25 mM) Sirt5^−/−^ ADMSCs under basal conditions and after 12 h of H_2_O_2_ (200 μM) treatment. G, Mean fluorescent intensity of DCFH‐DA staining (n = 3). H, Oxygen consumption rates (OCRs) of WT ADMSCs, Sirt5^−/−^ ADMSCs, and glycine‐treated (25 mM) Sirt5^−/−^ ADMSCs. I, Extracellular acidification rates (ECARs) of WT ADMSCs, Sirt5^−/−^ ADMSCs, and glycine‐treated (25 mM) Sirt5^−/−^ ADMSCs. J, ECAR/OCR ratios. ^#^ *P* < .05 versus glycine‐treated Sirt5^−/−^ ADMSCs, ^##^ *P* < .01 versus glycine‐treated Sirt5^−/−^ ADMSCs, ^*^ *P* < .05 versus WT, ^**^ *P* < .01 versus WT (n = 4). K, Relative mRNA expression levels of glycolysis‐related genes (n = 6). L, Relative mRNA expression levels of oxidative (*G6pd, Pgls, Pgd*) and nonoxidative (*Rpia, Tkt, Rpe*) pentose phosphate pathway‐related genes (n = 6). Data are expressed as the mean ± SD. ^*^ *P* < .05; ^**^ *P* < .01; ^***^ *P* < .001; ^****^ *P* < .0001

Glycine has been shown to reduce protein succinylation by removing succinyl‐CoA.^27^ Therefore, we aimed to determine if glycine treatment could reverse the previously observed increased proliferation rate of Sirt5^−/−^ ADMSCs via inhibition of succinylation. Indeed, SIRT5 deficiency‐induced hypersuccinylation was successfully blocked by glycine treatment in a dose‐dependent manner (Figure 6A), and a CCK8 cell viability assay showed that the self‐renewal capacities of Sirt5^−/−^ ADMSCs were attenuated along with these decreased succinylation levels (Figure 6B). Similarly, a cell counting assay confirmed a decreased proliferation rate of glycine‐treated Sirt5^−/−^ ADMSCs compared with WT and untreated Sirt5^−/−^ ADMSCs (Figure 6C). H_2_O_2_ induced ROS accumulation was augmented in glycine‐treated Sirt5^−/−^ ADMSCs (Figure 6D,E). This impaired anti‐oxidant capacity was further confirmed by DCFH‐DA staining‐based flow cytometry (Figure 6F,G). Oxygen consumption rate was not drastically altered by glycine supplement in Sirt5^−/−^ ADMSCs (Figure 6H). However, ECAR was declined in glycine‐treated Sirt5^−/−^ ADMSCs compared to WT and untreated Sirt5^−/−^ ADMSCs (Figure 6I). In Sirt5^−/−^ ADMSCs, metabolism predominantly occurred via glycolysis compared with WT ADMSCs, as previously suggested (Figure 6J). Nonetheless, glycine treatment reversed the previously observed metabolic pattern of Sirt5^−/−^ ADMSCs (Figure 6J), which resulted in the redirection of glucose metabolism to mitochondrial respiration. Furthermore, the mRNA expression levels of glycolysis‐related enzymes were significantly attenuated in glycine‐treated Sirt5^−/−^ ADMSCs compared with untreated Sirt5^−/−^ ADMSCs (Figure 6K). A slight reduction in the expression levels of PPP‐related enzymes was also detected; however, there was no significant difference between glycine‐treated and untreated Sirt5^−/−^ ADMSCs (Figure 6L).

### 3.7 Sirt5 re‐expression reversed metabolic pattern and proliferative phenotype of Sirt5‐knockout ADMSCs

**FIGURE 7:**
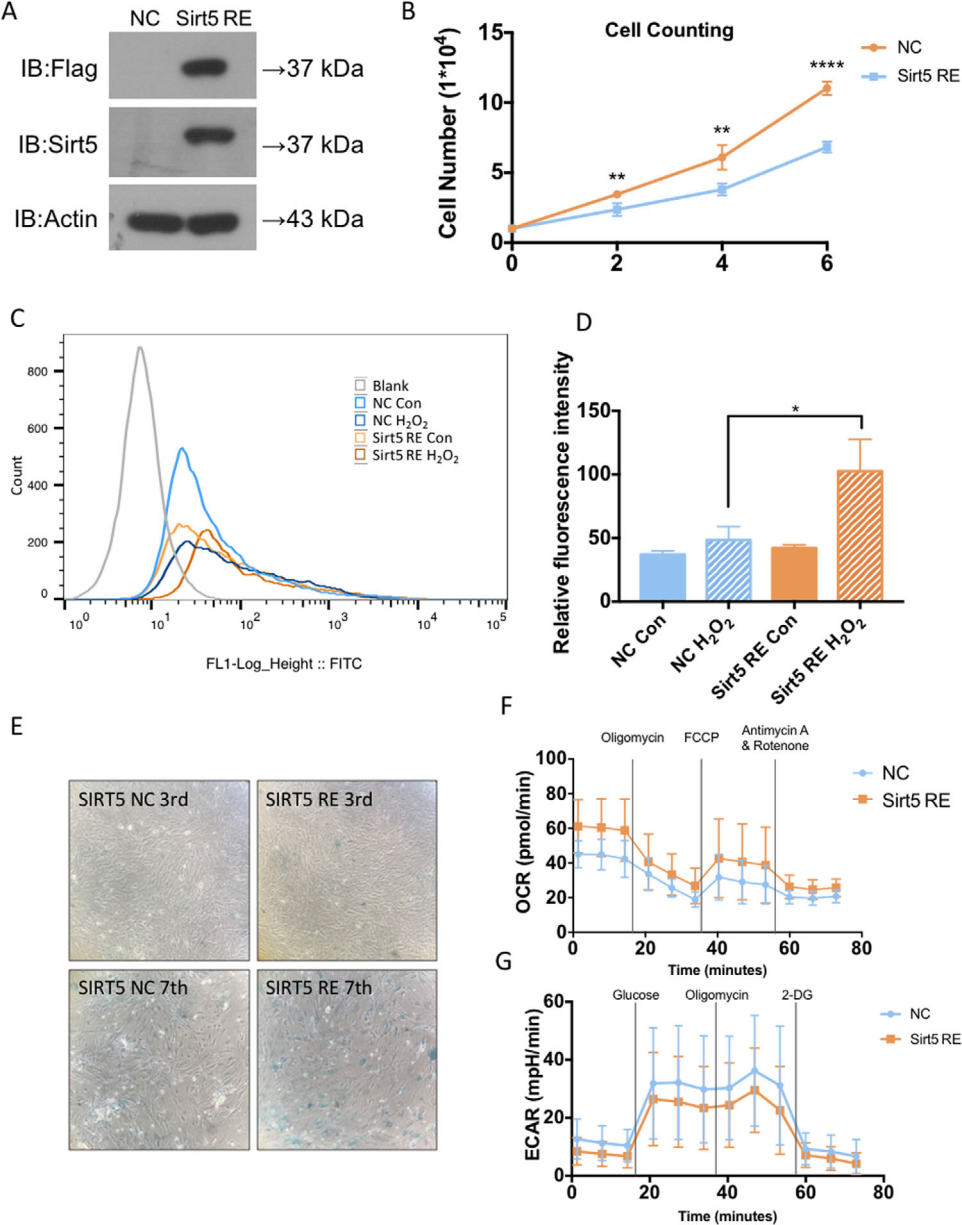
Sirt5 re‐expression reversed metabolic pattern and proliferative phenotype of Sirt5‐knockout ADMSCs: A, Protein expression levels of SIRT5 in control virus transfected Sirt5^−/−^ ADMSCs (NC) and Sirt5 overexpression virus transfected Sirt5^−/−^ ADMSCs (Sirt5 RE). B, Cell counting assay of NC and Sirt5 RE ADMSCs at days 2, 4, and 6 after seeding (n = 4). C, Representative flow cytometry assay image (DCFH‐DA staining) of NC and Sirt5 RE ADMSCs. D, Mean fluorescent intensity of DCFH‐DA staining (n = 3). E, Senescence‐associated β‐galactosidase (SA‐β‐gal) staining (blue) of NC and Sirt5 RE ADMSCs at passages 3 and 7 (upper panel: passage 3; lower panel: passage 7). F, Oxygen consumption rates (OCRs) of NC and Sirt5 RE ADMSCs (n = 6). G, Extracellular acidification rates (ECARs) of NC and Sirt5 RE ADMSCs (n = 6). Data are expressed as the mean ± SD. ^*^ *P* < .05; ^**^ *P* < .01; ^***^ *P* < .001; ^****^ *P* < .0001

To further elucidate regulatory effects of SIRT5 in ADMSCs during in vitro expansion, Sirt5 was re‐expressed by lentiviral transfection in Sirt5^−/−^ADMSCs (Figure 7A). Re‐expression of SIRT5 protein dramatically slowed the growth of Sirt5^−/−^ ADMSCs (Figure 7B). Anti‐oxidant capacity was attenuated in SIRT5 re‐expression ADMSCs (SIRT5 RE) compared to control Sirt5^−/−^ ADMSCs (NC), as the mean fluorescence intensity of DCFH‐DA staining was significantly increased in Sirt5 RE group after H_2_O_2_ treatment (Figure 7C,D). Additionally, SIRT5 recovery also led to accelerated cellular senescence (Figure 7E). Moreover, elevated oxidative phosphorylation with decreased glycolysis activity were observed in Sirt5 RE cells (Figure 7F,G), revealing a reversed metabolic pattern after Sirt5 re‐expression. Taken over, these data indicated that Sirt5 re‐expression reversed metabolic pattern and proliferative phenotype of Sirt5^−/−^ ADMSCs, further confirm the benefit effects of Sirt5 deficiency in cultured ADMSCs.

### 3.8 Local injection of Sirt5‐knockout ADMSCs enhanced blood flow recovery and angiogenesis in the hind limb ischemia mouse model

**FIGURE 8:**
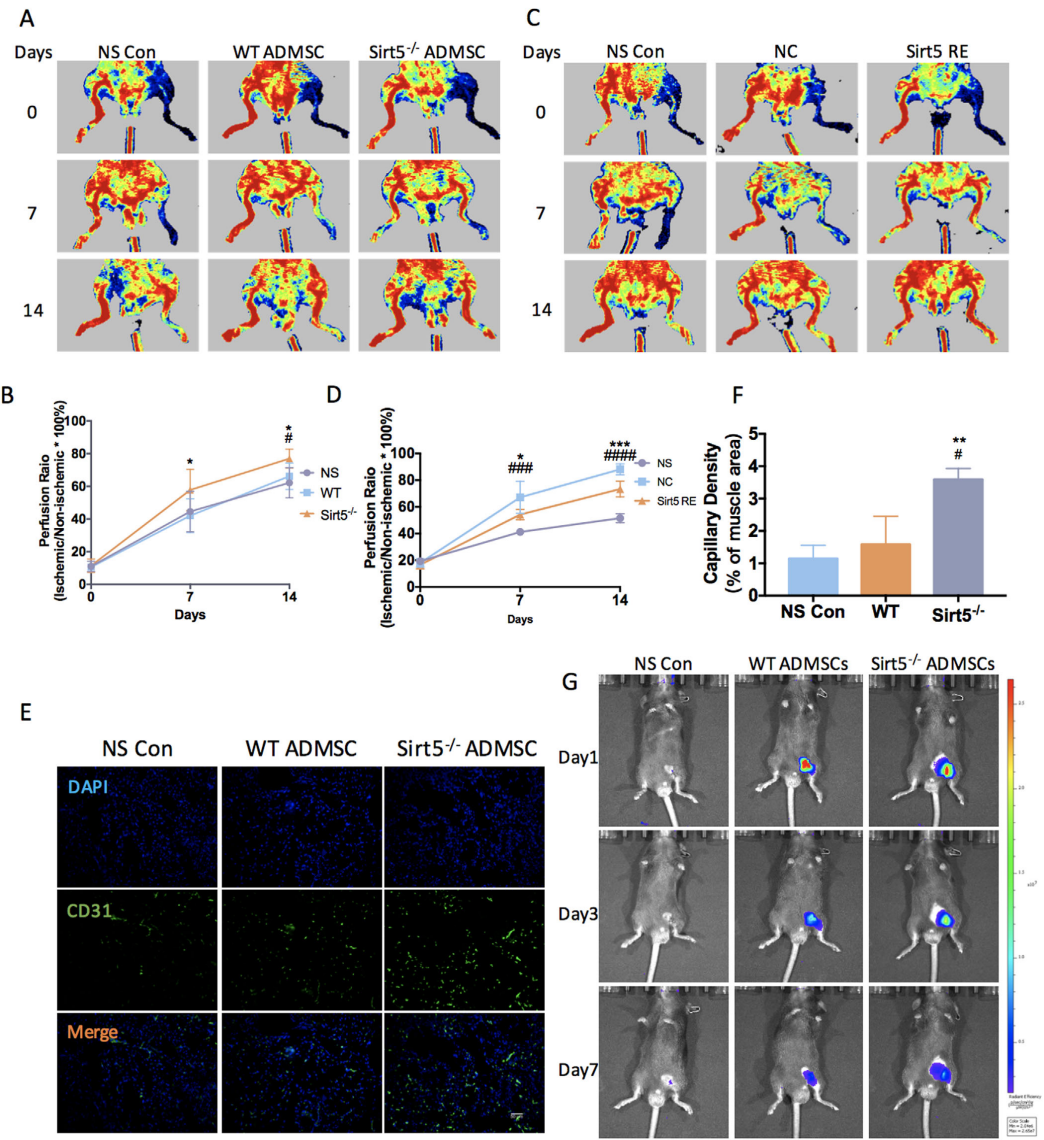
Local injection of Sirt5‐knockout ADMSCs enhanced blood flow recovery and angiogenesis in the hind limb ischemia model. A, Laser Doppler perfusion imaging of the hind limb ischemia mouse model treated with NS, WT ADMSCs or Sirt5^−/−^ ADMSCs on days 0, 7, and 14. ^*^ *P* < .05 versus normal saline (NS) control, ^**^ *P* < .01 versus NS control, ^#^ *P* < .05 versus wild‐type (WT). B, Perfusion ratio quantification in the ischemic hind limb treated with NS, WT ADMSCs or Sirt5^−/−^ ADMSCs (n = 6). Perfusion ratios were calculated as the ischemic limbs versus the non‐ischemic limbs in each group. C, Laser Doppler perfusion imaging of the hind limb ischemia mouse model treated with NS, control lentivirus transfected (NC) or Sirt5 overexpression lentivirus transfected (Sirt5 RE) ADMSCs on days 0, 7, and 14. ^###^ *P* < .001 versus normal saline (NS) control; ^####^ *P* < .0001 versus NS control; ^*^ *P* < .05 versus Sirt5 RE; ^***^ *P* < .001 versus Sirt5 RE. D, Perfusion ratio quantification in the ischemic hind limb treated with NS, NC, or Sirt5 RE (n = 5). E, CD31 immunofluorescence staining in the quadriceps femoris of the ischemic hind limbs (Green, CD31; Blue, DAPI). Scale bar: 50 μm. F, Capillary density quantification (n = 3). G, In vivo fluorescence imaging of ischemic hind limb treated with DiR Iodide labeled NC and Sirt5 RE ADMSCs on days 0, 3, and 7

We demonstrated that Sirt5^−/−^ ADMSCs exhibited sustained stemness and proliferative qualities as well as reduced ROS accumulation compared with WT ADMSCs, all of which suggest an improved therapeutic potential of Sirt5^−/−^ ADMSCs. Therefore, we used a hind limb ischemia model to test the therapeutic functions of WT and Sirt5^−/−^ ADMSCs. After femoral artery ligation, the ischemic limbs had an average profusion rate of approximately 10% compare with that of the non‐ischemic limbs in all three groups (Sirt5^−/−^ ADMSC‐treated, WT ADMSC‐treated, and control NS‐treated groups; Figure 8A,B). On day 7 post‐ligation, the Sirt5^−/−^ ADMSC‐treated group showed accelerated blood flow recovery compared with the WT ADMSC‐treated group (Sirt5^−/‐^ vs WT: 57.75% vs 42.02%). This enhanced therapeutic effect was further observed on day 14 post‐ligation, when the perfusion rate of the Sirt5^−/−^ ADMSC‐treated ischemic limbs was approximately 10% higher than that of the WT ADMSC‐treated or NS‐treated ischemic limbs. Compared with the NS control group, WT ADMSC injection only led to a slight, but not significant, elevation in the perfusion rate (WT vs NS: 66.2% vs 62.1%. *P* = .4545). However, re‐expression of SIRT5 in Sirt5^−/−^ ADMSCs eliminated their enhanced therapeutic effect in ischemic limbs (Figure 8C). Consistent with the blood flow recovery rate, treatment with Sirt5^−/−^ ADMSCs also exhibited improved angiogenesis, which was detected by CD31 staining, compared with the other groups (Figure 7C,D). Moreover, the retention after transplantation of these cells was determined using in vivo tracking. SIRT5 deficiency increased the ratio of survival ADMSCs (DiR Iodide labeled) compared to WT ADMSCs (Figure 8G). Thus, these hind limb ischemia model data showed an improved *in vivo* therapeutic capacity of Sirt5^−/−^ ADMSCs compared with WT ADMSCs.

## 4 DISCUSSION

Prolonged in vitro expansion of MSCs leads to the loss of their stemness and therapeutic potential.^5^ In this study, we found that SIRT5 gradually accumulates and exerts metabolic regulatory effects in MSCs during in vitro culture. Knockout of SIRT5 in ADMSCs results in the hypersuccinylation of TCA cycle enzymes, including SDHA, MDH2, and OGDH, thus suppressing mitochondrial respiration and directing glucose flux to glycolysis and the PPP. This metabolic switch in Sirt5^−/−^ ADMSCs contributes to enhanced proliferation, decreased ROS accumulation, and improved therapeutic functions, as shown in our proposed mechanistic model of ischemic disease (Figure 8). These findings may provide additional insights into strategies for improving the quality of MSCs for successful clinical application.

SIRT5 has been previously reported to be a master metabolic regulator, and its expression levels have been significantly associated with metabolic patterns and cellular functions in various cell types. The metabolism of proliferative cells is extremely important in determining cell fate and function.^9^ Under in vivo conditions, MSCs occupy stem cell niches with relatively hypoxic microenvironments. HIF‐1α is stabilized within these microenvironments, thus activating the transcription of glycolysis‐related genes and glucose transporters. However, under normoxic conditions, HIF‐1α easily undergoes proteasome degradation. MSCs have the ability to maintain in vitro HIF‐1α levels via elevated transcription rates^28^ to maintain aerobic glycolysis. Aerobic glycolysis is a hallmark of stemness and is crucial for survival in many types of stem cells, including MSCs. Nonetheless, MSCs gradually lose the ability to maintain aerobic glycolysis and high levels of HIF‐1α transcription during in vitro expansion, which leads to metabolic reprogramming and a reduction in the therapeutic efficacy of the MSCs.^11^, ^29^ HIF‐1α stabilization is an effective way to maintain the cellular functions of MSCs during standard culture.^30^, ^31^ Selak et al reported that succinate accumulation due to SDH inhibition leads to HIF‐1α stabilization by suppressing HIF‐1α hydroxylases.^32^ We found that SIRT5 deficiency in ADMSCs results in attenuation of the TCA cycle via hypersuccinylation of its enzymes and suppresses mitochondrial function, which mimics in vivo stem cell conditions. Therefore, SDHA hypersuccinylation due to SIRT5 deficiency may also lead to HIF‐1α stabilization in ADMSCs. Sirt5^−/−^ ADMSCs also exhibit upregulated levels of GLUT1 and HIF‐1α, resulting in augmented glycolysis and metabolism via the PPP to provide the energy required for proliferation as well as a source of reductants for antioxidant purposes. Altogether, our results suggest that SIRT5 deficiency in ADMSCs attenuates mitochondrial respiration and ROS accumulation, increases HIF‐1α levels, and reverses the metabolic pattern that occurs during in vitro expansion to one that better mimics that of cells within the in vivo microenvironment.

SIRT5 is characterized as a unique desuccinylase, demalonylase, and deglutarylase with weak deacetylase activity. The exact regulatory effects of SIRT5 are highly context specific in certain types of cells, which may explain the discrepancies in the types and sites of posttranslational modifications. However, proteins of the TCA cycle are common downstream targets of SIRT5 that are regulated by lysine succinylation,^16^, ^33^ indicating the close relationship between SIRT5 and glucose metabolism. Li et al reported that hypersuccinylation of isocitrate dehydrogenase 1 (IDH1), pyruvate dehydrogenase E1 component subunit alpha (PDHA), succinate dehydrogenase complex iron‐sulfur subunit B (SDHB), and cyclooxygenase (COX) attenuates their catalytic activities, thus leading to impaired mitochondrial respiration and an enhanced Warburg effect in U87MG glioblastoma cells.^27^ Similarly, specific knockout of SIRT5 in the brown adipose tissues (BATs) of mice results in hypersuccinylation of SDHA and SDHB as well as decreased OCRs compared with WT BATs, also indicating reduced mitochondrial respiration.^21^ Consistent with these findings, our findings suggest a critical regulatory role of SIRT5 in the TCA cycle. The most highly succinylated TCA enzymes in Sirt5^−/−^ ADMSCs are MDH2, SDHA, and OGDH. Notably, OGDH is a rate‐limiting enzyme that largely determines the flux rate of the entire TCA cycle. Therefore, hypersuccinylation and catalytic inhibition of these enzymes collectively leads to suppression of the TCA cycle and attenuation of mitochondrial respiration. In several studies, SIRT5 were reported to enhance glycolysis and negatively regulate TCA activity.^16^, ^18^ This inconsistency may due to the distinct predominant acylation types in different studies. Park et al revealed an increase in SDH activity in SIRT5 KD HEK293T cells. However, the exact lysine succinylation sites of SDHA were different between Park's study and the current study (reference^16^ and Figure 4B), leading to contrary effects on SDH activity. Though functions of SIRT5 are cell‐ and context‐specific, these studies all confirmed SIRT5 as a master metabolic regulator.

In this study, we found that SIRT5 deficiency leads to attenuated TCA cycle activity but upregulated glycolysis and metabolism via the PPP. Numerous studies have reported the regulatory effects of SIRT5 in glycolysis; however, few of them have provided direct evidence of its effects in the PPP. Gao et al revealed that SIRT5 could active PPP through triosephosphate isomerase demalonylation. However, proteins malonylation levels were similar in WT and Sirt5^−/‐^ ADMSCs. Therefore, elevated^34^ metabolism through PPP in Sirt5^−/‐^ ADMSCs may due to increased glucose uptake and enhanced aerobic glycolysis. The percentage of glucose metabolized by the PPP varies from 5% to 30% and largely depends on cell conditions. The PPP is the central source of nucleic acid precursors and reducing equivalents for proliferating cells. G6PD sets the pace of the PPP and is the key enzyme for NADPH production.^26^ Many studies have demonstrated that an enhanced PPP is associated with tumor cell proliferation, metastasis, and cancer recurrence.^35^ Moreover, NADPH provided by the PPP protects certain tumor cells against chemotherapy‐induced ROS accumulation and cell death.^36^ In Sirt5^−/−^ ADMSCs, the expression of G6PD and other PPP‐related enzymes are upregulated at both the mRNA and protein levels. This may account for the proliferative phenotype and reduced ROS accumulation under oxidative stress of Sirt5^−/−^ ADMSCs, which ultimately leads to their preserved therapeutic functions in the hind limb ischemia model. In addition to G6PD and other enzymes in the oxidative branch of the PPP, enzymes within the nonoxidative branch have also been suggested to be important sources for ribosome 5‐phosphate production in tumor cells.^37^, ^38^ All metabolic reactions are dynamic and reversible, and the nonoxidative flux is determined by the levels of fructose 6‐phosphate and/or glyceraldehyde 3‐phosphate, which are regulated by 6‐phosphofructokinase 1 (PFK1).^39^ Consistently, we found that nonoxidative enzymes, including TKT, RPIA, RPE, and PFK1, are upregulated in Sirt5^−/−^ ADMSCs compared with WT ADMSCs.

MSCs have been explored for many years as a treatment for ischemic diseases; however, the clinical results are inconsistent. One possible explanation is poor in vivo survival of the transplanted MSCs. In MSCs, metabolic patterns that change in response to cellular conditions greatly contribute to cell fate. It was reported that elevated glycolysis, HIF‐1α expression, and glucose uptake, along with attenuated OXPHOS, leads to improved MSC survival under ischemic conditions.^29^ In this study, SIRT5 deficiency induces a metabolic switch that promotes the survival of ADMSCs in the hind limb ischemia model. Additionally, Sirt5^−/−^ ADMSCs show enhanced resistance to oxidative stress. Therefore, these Sirt5‐deficiency benefits may be responsible for the observed therapeutic improvements.

Despite these impactful findings, our study has a limitation. During ADMSC in vitro culture, *Sirt2* mRNA levels are also significantly altered between early and late passages. In a variety of stem cells, the role of SIRT2 in cellular metabolic processes has been intensively investigated.^25^, ^40^ SIRT5 was also proved as a master metabolic regulator in other cells. However, little is known about the role of SIRT5 in stem cell metabolism. Therefore, the current study aims to clarify the functional effects of SIRT5 on ADMSCs during expansion. Although the metabolic regulatory effect of SIRT2 in other stem cells has been previously reported, its effect in in vitro cultured ADMSCs is not elucidated in this study and warrants further investigation.

In conclusion, our study revealed that SIRT5 deficiency in cultured ADMSCs switches their metabolic pattern to a state that more resembles that of the cells in the in vivo microenvironment, which leads to enhanced self‐renewal capacities and improved therapeutic functions for ischemic disease treatment. Metabolic programming has emerged as an important factor between large‐scale MSC bio‐manufacturing and successful clinical outcomes. Metabolism modulation is an effective method to enhance the functional properties of MSCs.^5^ Our findings may provide a potential novel strategy to maximize MSC yield while preserving their therapeutic functions, thus filling the gap between maturing theory and limited clinical outcomes of mesenchymal stem cell therapy.

## 5 CONCLUSIONS

We demonstrated that SIRT5 accumulates in ADMSCs during continuous in vitro passaging and is associated with a loss of stemness. SIRT5 deficiency in ADMSCs reverses in vitro metabolic reprogramming to a more endogenous metabolic pattern by elevating aerobic glycolysis and attenuating mitochondrial respiration. Furthermore, these metabolic alterations in Sirt5^−/−^ ADMSCs enhance their proliferative and therapeutic functions. Our results demonstrate the role of SIRT5 and its metabolic regulation in cultured ADMSCs. Additionally, we advocate SIRT5 as a potential target to improve the quality of ADMSCs for clinical application. Our findings may not only benefit MSC therapies, but may also provide insight into strategies for other therapies using stem cells with similar metabolic patterns.

## AUTHOR CONTRIBUTIONS

TO, WY, HZ, HL, AS, and JG designed the experiments; TO, WY, WL, YL, ZD, YL, and ZQ performed the experiments and collected data; TO, WY, SC, and XS analyzed data; XS, XW, ZD, SC, and KH revised the manuscript; TO, WY, and AS wrote the manuscript; AS and JG supervised the study.

## CONFLICT OF INTEREST

The authors declare no conflict of interest.

## Supporting information

SUPPORTING INFORMATION

SUPPORTING INFORMATION

**Supplementary Figure 1. Stem cell surface and multipotency marker levels of isolated ADMSCs**. (A) Stem cell surface marker expression levels of wild‐type (WT) and Sirt5‐knockout (Sirt5^−/−^) ADMSCs (upper panel: CD29, CD10; lower panel: SCA‐1, CD45). Gray: blank; Blue: WT ADMSCs; Orange: Sirt5^−/−^ ADMSCs. (B) Alizarin Red S staining of osteocytes (upper panel) and Oil Red O staining of adipocytes (lower panel) for confirmation of osteogenesis and adipogenesis, respectively, in WT and Sirt5^−/−^ ADMSCs. Scale bar: 200 μm. (C) Protein expression levels of SIRT5 in wild‐type (WT) and Sirt5‐knockout (Sirt5^−/−^) ADMSCs.

**Supplementary Figure 2. Population doubling time and aging‐related marker expressions in WT and Sirt5 knockout ADMSCs** (A) Population doubling time of wild‐type (WT) and Sirt5‐knockout (Sirt5^−/−^) ADMSCs (n = 5). (B) Relative mRNA expression levels of aging‐related markers and senescence associated secretory phenotypes (SASPs) (n = 6). Data are expressed as the mean ± SD. *P < 0.05, **P < 0.01, ***P < 0.001, ****P < 0.0001.

Supplementary Figure 3. Quantification of glycolysis‐ and pentose phosphate pathway‐related protein expression levels in wild‐type and Sirt5‐knockout ADMSCs. (A) Quantification of glycolysis‐related protein expression levels in wild‐type (WT) and Sirt5‐knockout (Sirt5^−/−^) ADMSCs (n = 3). (B) Quantification of pentose phosphate pathway‐related protein expression levels in WT and Sirt5^−/‐^ ADMSCs. Data are expressed as the mean ± SD. *P < 0.05, **P < 0.01. β‐actin was used as a reference gene.

## Data Availability

The dataset used during this study is available from the corresponding author upon reasonable request.
