# Supplementary material for: SIRT5 deficiency enhances the proliferative and therapeutic capacities of adipose‐derived mesenchymal stem cells via metabolic switching
Source: Clin Transl Med. 2020 Sep 23;10(5):e172. doi: 10.1002/ctm2.172 (PMC7510333; doi:10.1002/ctm2.172)
Supplement: Supplementary file 1 — SUPPORTING INFORMATION [file CTM2-10-e172-s001.docx]

Supplementary Experiment Protocols

**1. Quantitative PCR**

The sequences of the primers used are as follows:

β-actin: (forward) 5’-GGCTGTATTCCCCTCCATCG-3’, (revers) 5’-CCAGTTGGTAACAATGCCATGT-3’;

Sirt1: (forward) 5’-GCTGACGACTTCGACGACG-3’, (reverse) 5’-TCGGTCAACAGGAGGTTGTCT-3’;

Sirt2: (forward) 5’-GCCTGGGTTCCCAAAAGGAG-3’, (reverse) 5’-GAGCGGAAGTCAGGGATACC-3’;

Sirt3: (forward) 5’-GAGCGGAAGTCAGGGATACC-3’, (reverse) 5’-CAACATGAAAAAGGGCTTGGG-3’;

Sirt4: (forward) 5’-GTGGAAGAATAAGAATGAGCGGA-3’, (reverse) 5’-GGCACAAATAACCCCGAGG-3’;

Sirt5: (forward) 5’-CTCCGGGCCGATTCATTTCC-3’, (reverse) 5’-GCGTTCGCAAAACACTTCCG-3’;

Sirt6: (forward) 5’-ATGTCGGTGAATTATGCAGCA-3’, (reverse) 5’-GCTGGAGGACTGCCACATTA-3’;

Sirt7: (forward) 5’-AGCATCACCCGTTTGCATGA-3’, (reverse) 5’-GGCAGTACGCTCAGTCACAT-3’;

Nanog: (forward) 5’-TCTTCCTGGTCCCCACAGTTT-3’, (reverse) 5’-GCAAGAATAGTTCTCGGGATGAA-3’;

Oct4: (forward) 5’-GGCTTCAGACTTCGCCTCC-3’, (reverse) 5’-AACCTGAGGTCCACAGTATGC-3’;

p53: (forward) 5’- GCGTAAACGCTTCGAGATGTT-3’, (reverse) 5’- TTTTTATGGCGGGAAGTAGACTG-3’

p21: (forward) 5’-CATCAGCCATGACGAGCTGTT-3’, (reverse) 5’-CTCTGTTCTACTGACCATCTTGC-3’,

p16: (forward) 5’-CGCAGGTTCTTGGTCACTGT-3’, (reverse) 5’-TGTTCACGAAAGCCAGAGCG-3’,

Tnf-a: (forward) 5’-GACGTGGAACTGGCAGAAGAG-3’, (reverse) 5’-TTGGTGGTTTGTGAGTGTGAG-3’,

Il-1b: (forward) 5’-TTCAGGCAGGCAGTATCACTCATTG-3’, (reverse) 5’-ACACCAGCAGGTTATCATCATCATCC-3’,

Il6: (forward) 5’-TAGTCCTTCCTACCCCAATTTCCC-3’, (reverse) 5’-TTGGTCCTTAGCCACTCCTTC-3’,

Mmp3: (forward) 5’-ACATGGAGACTTTGTCCCTTTTG(reverse)-3’, 5’-TTGGCTGAGTGGTAGAGTCCC-3’,

Vegf: (forward) 5’-GCACATAGAGAGAATGAGCTTCC-3’, (reverse) 5’-CTCCGCTCTGAACAAGGCT-3’;

Hgf: (forward) 5’-ATGTGGGGGACCAAACTTCTG-3’, (reverse) 5’-GGATGGCGACATGAAGCAG-3’;

Igf: (forward) 5’-CTGGACCAGAGACCCTTTGC-3’, (reverse) 5’-GGACGGGGACTTCTGAGTCTT-3’;

Glut1: (forward) 5’-GCAGTTCGGCTATAACACTGG-3’, (reverse) 5’-GCGGTGGTTCCATGTTTGATTG;

Pkm: (forward) 5’-TGGTGACGGAGGTGGAGAATGG-3’, (reverse) 5’-GTCGGCTGCCTTGCGGATG

Hif-1α: (forward) 5’-ACCTTCATCGGAAACTCCAAAG-3’, (reverse) 5’-ACTGTTAGGCTCAGGTGAACT;

Hk1: (forward) 5’-CCTCCGTCAAGATGCTGCCAAC-3’, (reverse) 5’-CCGCCGAGATCCAGTGCAATG;

Pfk1: (forward) 5’-GCATCAAGCAGTCAGCCTCAGG-3’, (reverse) 5’-AGCCAGGTAGCCACAGTAGCC;

Pfkfb3: (forward) 5’-CAACGCCTGGAGCCTGTGATC-3’, (reverse) 5’-CAGCGTAGAACAGCCTGGTGAC

G6pd: (forward) 5’-CACAGTGGACGACATCCGAAA-3’, (reverse) 5’-AGCTACATAGGAATTACGGGCAA;

Pgls: (forward) 5’-CCAGGTCCTTACCATCAATCCT-3’, (reverse) 5’-AGGGAAGAGCGAACAGGTATG;

Pgd: (forward) 5’-TGAAGGGTCCTAAGGTGGTCC-3’, (reverse) 5’-CCGCCATAATTGAGGGTCCAG;

Rpia: (forward) 5’-AAGGCCGAGGAGGCTAAGAA-3’, (reverse) 5’-CTTTCAGCTATTCGCTGCACA;

Rpe: (forward) 5’-TGATGACCCCAGAGCTGTTAT-3’, (reverse) 5’-GTGGCTTCCTCGATCAAGGG;

Tkt: (forward) 5’-ATGGAAGGTTACCATAAGCCAGA-3’, (reverse) 5’-TGCAGCATGATGTGGGGTG.

**2. Population doubling time (PDT)**

Cells were detached with 0.25% trypsin, suspended in stem cell basal medium, and counted using a hemocytometer. An equal number of cells (3000) was seeded into the individual wells of 96-well plates. The populations of cells were measured by CCK8 (Beyotime, Shanghai, China C0037) at 0h and 48h post seeding. Population doubling time was calculated using following formula: PDT=48×lg2/(lgN48-lgN0). N48: OD450 value obtained after 48h; N0: OD450 value obtained after seeding.

**3. Immunochemistry and immunofluorescence assays**

For Ki67 staining, ADMSCs at passages 1, 3 and 7 were seeded into a 12-well plate. Cells were fixed in 4% paraformaldehyde for 15 minutes and permeabilized with 0.2% Triton X-100 for 5 minutes at room temperature. Next, the cells were incubated with Ki67 antibody (Abcam; ab15580) overnight at 4°C. Cells were then incubated with a fluorescent secondary antibody (Invitrogen, Thermo Fisher, Waltham, MA, USA; A32723) for 30 minutes at room temperature. Anti-fade DAPI solution was applied after secondary antibody incubation, and images were captured by a fluorescence camera (ZEISS Group, Jena, Germany). The percentage of positive cells was analyzed using ImageJ software (National Institutes of Health, Bethesda, MD, USA).

For dihydroethidium (DHE) staining, ADMSCs were seeded into a 6-well plate and cultured until 60–70% confluence. Before staining, ADMSCs in H_2_O_2_ groups were treated with 200 μM H_2_O_2_ for 12 hours. Cells were incubated with 5 μM DHE (Invitrogen) at 37°C for 40 minutes. Images were captured by a fluorescence camera, and fluorescence intensities were analyzed using ImageJ software.

For CD31 staining, mice subjected to hind limb ischemia were anesthetized and sacrificed 4 weeks after femoral artery ligation. Quadriceps femoris muscles were harvested, placed in a small box, and embedded with tissue-freezing medium. The box was frozen in liquid nitrogen, and the tissue block was cryosectioned at -20°C into 8-μm sections. The sections were placed on slides and allowed to dry for 1 hour. The slides were then fixed with 4% paraformaldehyde for 15 minutes, permeabilized with 0.2% Triton X-100 for 5 minutes at room temperature, and incubated with anti-CD31 antibody (Abcam; 24590) overnight at 4°C. Next, slides were incubated with a fluorescent secondary antibody for 30 minutes (Invitrogen; A32723) at room temperature. Anti-fade DAPI solution was applied after secondary antibody incubation, and images were captured by a fluorescence camera. Capillary densities were analyzed using ImageJ software.

**4. Liquid chromatography-mass spectrometry**

Liquid chromatography-mass spectrometry (LC-MS) was performed by PTM Biolab (Hangzhou, China). ADMSCs from Sirt5-knockout and littermate control C57BL/6 mice (20 mice per group) were subjected to succinylation affinity enrichment and subsequent LC-MS analysis. Samples were sonicated three times on ice in lysis buffer using a high-intensity ultrasonic processor. For digestion, the protein solutions were reduced with 5 mM dithiothreitol for 30 minutes at 56°C and alkylated with 11 mM iodoacetamide for 15 minutes at room temperature in the dark. Protein samples were then diluted by adding 100 mM triethylammonium bicarbonate (TEAB) to urea at a concentration less than 2 M. Finally, trypsin was added at a 1:50 trypsin-to-protein mass ratio for the first overnight digestion, and then a 1:100 trypsin-to-protein mass ratio was added for a second 4-hour digestion. To enrich modified peptides, tryptic peptides dissolved in NETN buffer were incubated overnight with pre-washed anti-succinyl lysine antibody conjugated with agarose beads (PTM Bio; PTM-402) at 4°C with gentle shaking. Then, the beads were washed four times with NETN buffer and twice with H_2_O. The bound peptides were eluted from the beads with 0.1% trifluoroacetic acid and vacuum dried. For LC-MS/MS analysis, the resulting peptides were desalted with C18 ZipTips (MilliporeSigma, Burlington, MA, USA). The tryptic peptides were dissolved in 0.1% formic acid with 2% acetonitrile (solvent A) and directly loaded into a home-made reversed-phase analytical column. The gradient was created using solvent B (0.1% formic acid in 98% acetonitrile) and consisted of an increase from 6–24% over 40 minutes, 24–38% over 12 minutes, and an eventual increase to 80% over 4 minutes, which was then held for the last 4 minutes. This was performed at a constant flow rate of 300 nL/minute on an EASY-nLC 1000 UPLC system. The peptides were subjected to nano-spray ionization (NSI) source followed by tandem mass spectrometry (MS/MS) in the Q Exactive Plus (Thermo Fisher) coupled online to ultra-performance liquid chromatography (UPLC). The applied electrospray voltage was 2.0 kV. The scan range was 350–1,550 m/z for full scan, and intact peptides were detected in the Orbitrap at a resolution of 60,000. Peptides were then selected for MS/MS using a normalized collision energy (NCE) setting of 28, and the fragments were detected in the Orbitrap at a resolution of 15,500. A data-dependent procedure that alternated between one MS scan followed by 20 MS/MS scans with 15.0-second dynamic exclusion was then performed. The automatic gain control (AGC) was set at 5e4 ion counts. The fixed first mass was set at 100 m/z, and the resulting MS/MS data were processed using the Maxquant search engine. Tandem mass spectra were searched against the mouse_SwissPort database, which was concatenated with a reverse decoy database. Trypsin/P was specified as a cleavage enzyme, allowing up to two missing cleavages. The mass tolerance for precursor ions was set to 20 ppm in the first search and 5 ppm in the main search, and the mass tolerance for fragment ions was set to 0.02 Da. Alkyl on cysteine was specified as a fixed modification, and lysine succinylation, N-terminal acetylation, and oxidation on MET were specified as variable modifications. FDR was adjusted to <1%.

**5. Functional Enrichment**

Enrichment of Gene Ontology analysis

Proteins were classified by GO annotation into three categories: biological process, cellular compartment and molecular function. For each category, a two-tailed Fisher’s exact test was employed to test the enrichment of the differentially expressed protein against all identified proteins. The GO with a corrected p-value < 0.05 is considered significant.

Enrichment of pathway analysis

Encyclopedia of Genes and Genomes (KEGG) database was used to identify enriched pathways by a two-tailed Fisher’s exact test to test the enrichment of the differentially expressed protein against all identified proteins. The pathway with a corrected p-value < 0.05 was considered significant. These pathways were classified into hierarchical categories according to the KEGG website.

Enrichment of protein domain analysis

For each category proteins, InterPro (a resource that provides functional analysis of protein sequences by classifying them into families and predicting the presence of domains and important sites) database was researched and a two-tailed Fisher’s exact test was employed to test the enrichment of the differentially expressed protein against all identified proteins. Protein domains with a p-value < 0.05 were considered significant.

**6. CCK8 cell viability assay**

Cells were seeded into a 96-well plate (3,000 cells/well). After culture for 24 hours, the culture medium was removed and replaced with 100 μL medium containing 10 μL CCK8 solution (Beyotime, Shanghai, China) per well, and the cells were then incubated for 1 hour at 37°C. Absorbance was measured at 450 nm using a microplate reader (Biotek).
