# Supplementary figures and images for: SIRT5 deficiency enhances the proliferative and therapeutic capacities of adipose‐derived mesenchymal stem cells via metabolic switching
Source: Clin Transl Med. 2020 Sep 23;10(5):e172. doi: 10.1002/ctm2.172 (PMC7510333; doi:10.1002/ctm2.172)

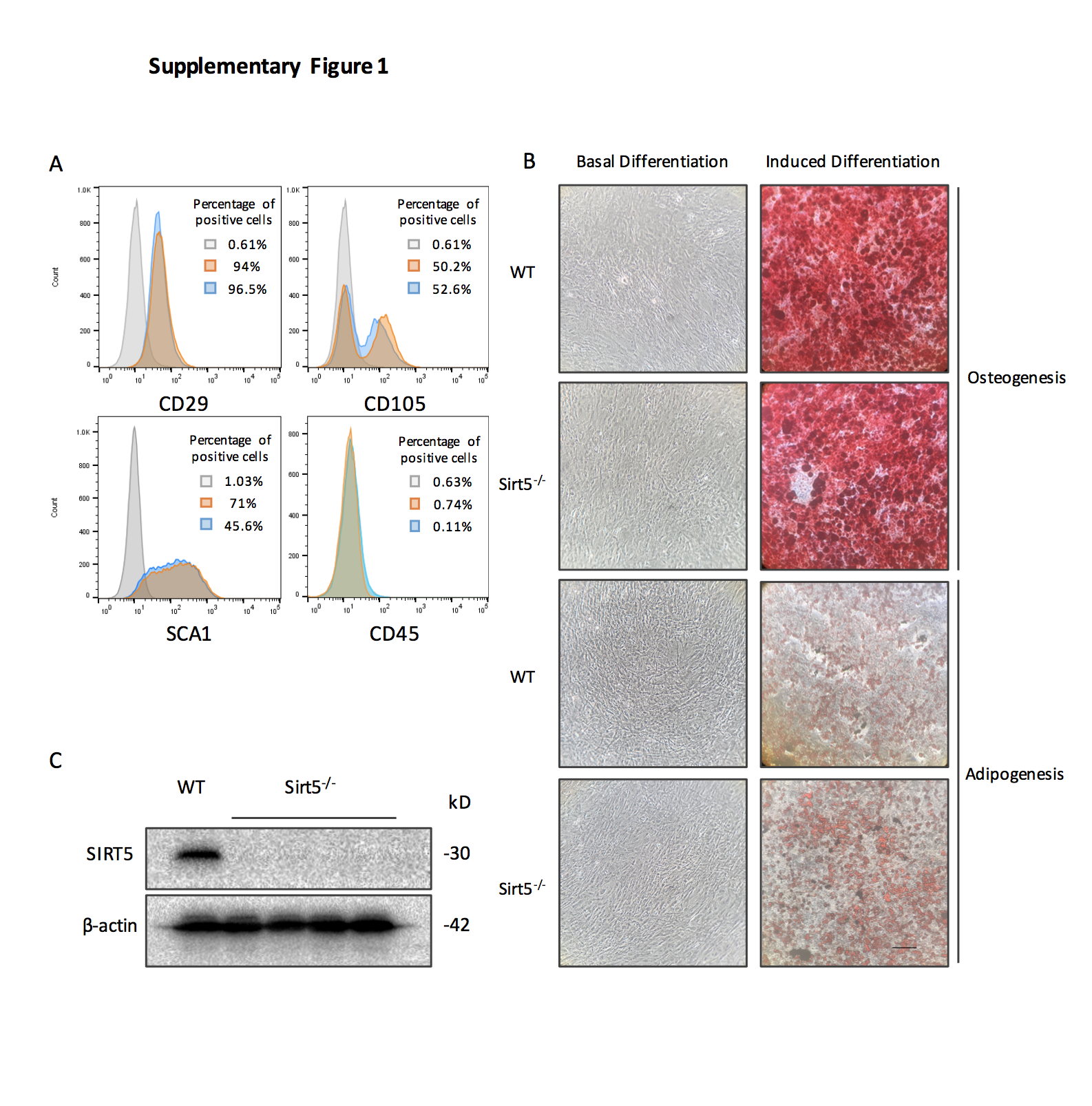

Supplement: Supplementary file 3 — Supplementary Figure 1. Stem cell surface and multipotency marker levels of isolated ADMSCs. (A) Stem cell surface marker expression levels of wild‐type (WT) and Sirt5‐knockout (Sirt5−/−) ADMSCs (upper panel: CD29, CD10; lower panel: SCA‐1, CD45). Gray: blank; Blue: WT ADMSCs; Orange: Sirt5−/− ADMSCs. (B) Alizarin Red S staining of osteocytes (upper panel) and Oil Red O staining of adipocytes (lower panel) for confirmation of osteogenesis and adipogenesis, respectively, in WT and Sirt5−/− ADMSCs. Scale bar: 200 μm. (C) Protein expression levels of SIRT5 in wild‐type (WT) and Sirt5‐knockout (Sirt5−/−) ADMSCs. [file CTM2-10-e172-s002.tif]

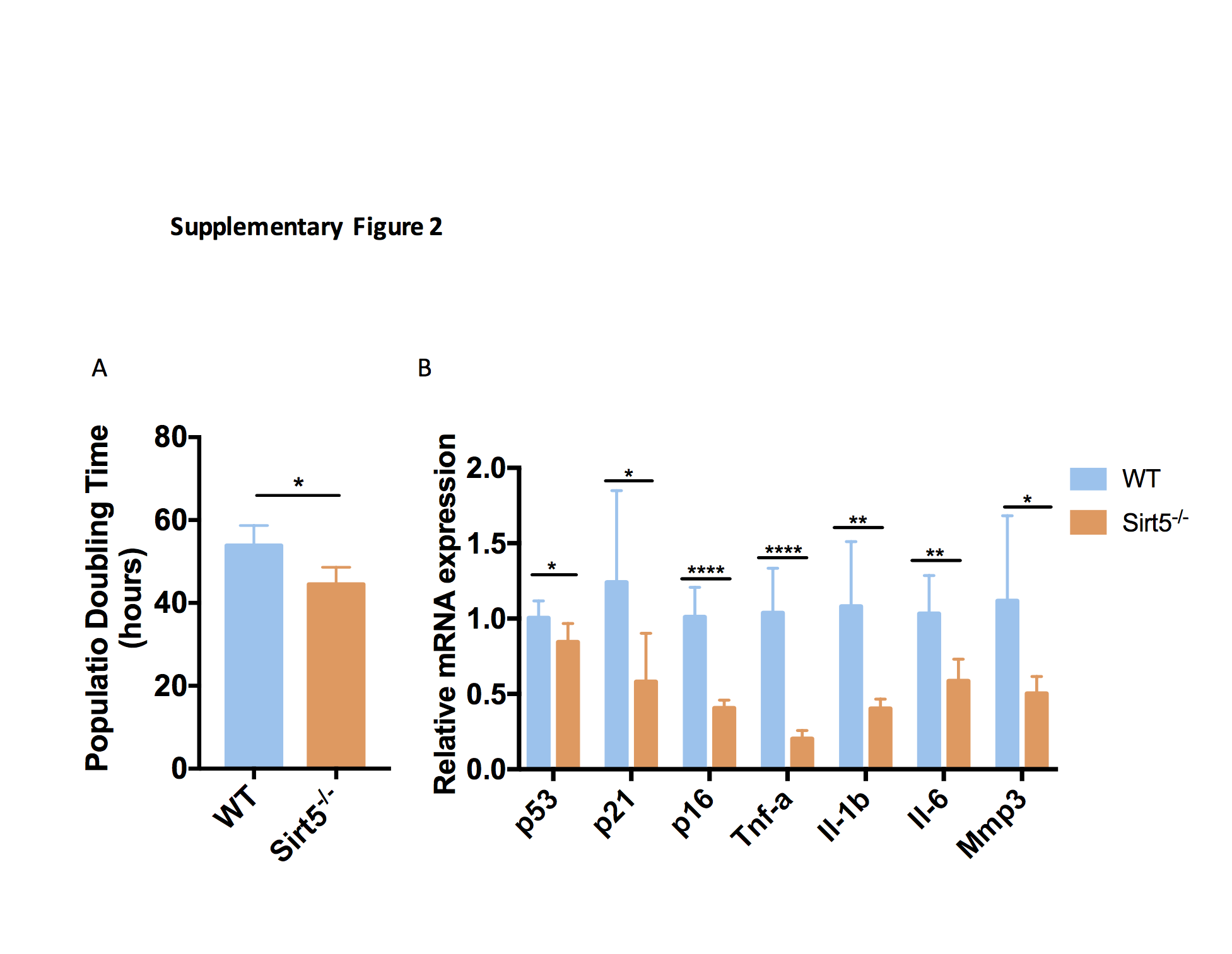

Supplement: Supplementary file 4 — Supplementary Figure 2. Population doubling time and aging‐related marker expressions in WT and Sirt5 knockout ADMSCs (A) Population doubling time of wild‐type (WT) and Sirt5‐knockout (Sirt5−/−) ADMSCs (n = 5). (B) Relative mRNA expression levels of aging‐related markers and senescence associated secretory phenotypes (SASPs) (n = 6). Data are expressed as the mean ± SD. *P < 0.05, **P < 0.01, ***P < 0.001, ****P < 0.0001. [file CTM2-10-e172-s003.tif]

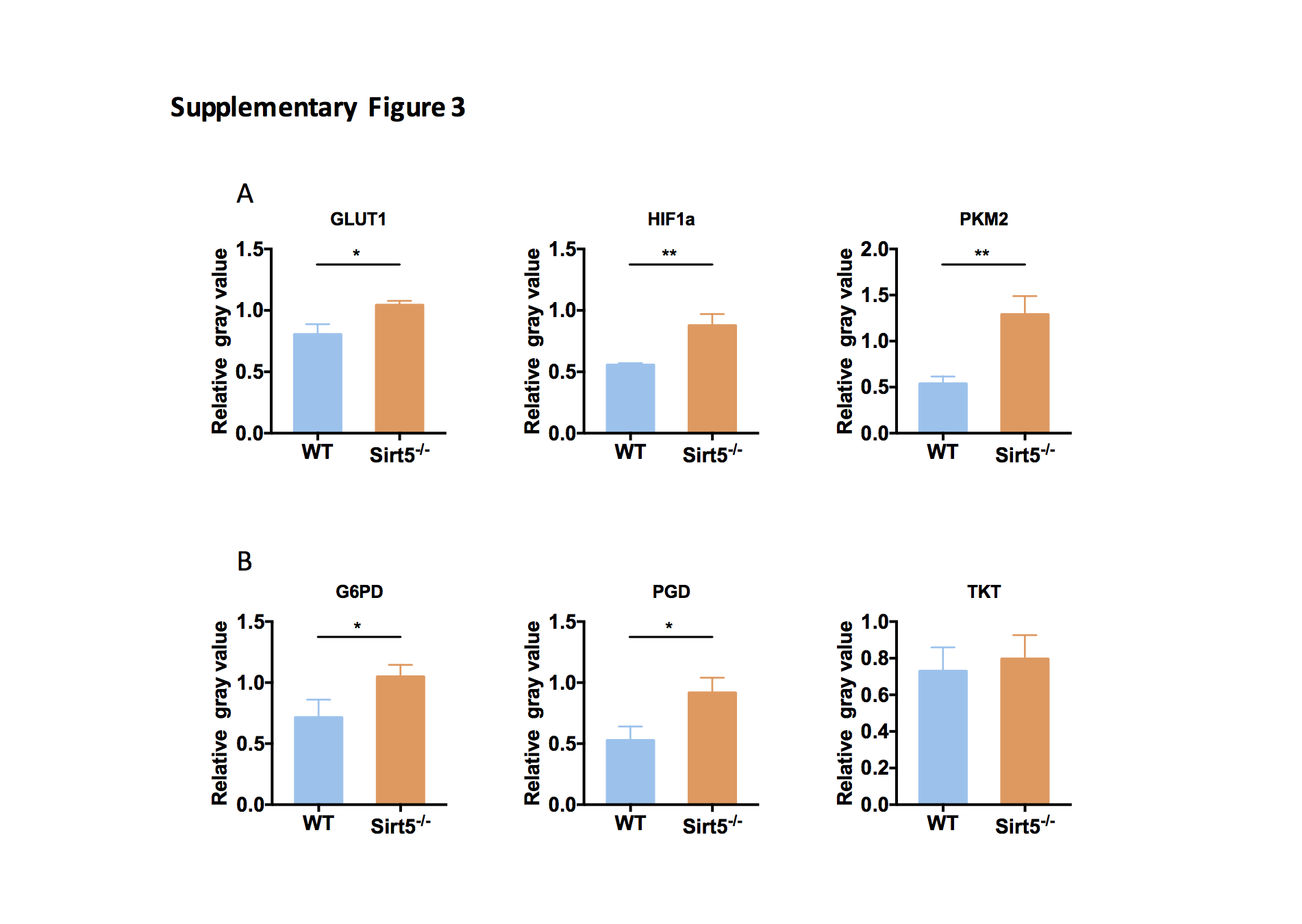

Supplement: Supplementary file 5 — Supplementary Figure 3. Quantification of glycolysis‐ and pentose phosphate pathway‐related protein expression levels in wild‐type and Sirt5‐knockout ADMSCs. (A) Quantification of glycolysis‐related protein expression levels in wild‐type (WT) and Sirt5‐knockout (Sirt5−/−) ADMSCs (n = 3). (B) Quantification of pentose phosphate pathway‐related protein expression levels in WT and Sirt5−/‐ ADMSCs. Data are expressed as the mean ± SD. *P < 0.05, **P < 0.01. β‐actin was used as a reference gene. [file CTM2-10-e172-s004.tif]
